# Advanced UAV–WSN System for Intelligent Monitoring in Precision Agriculture †

**DOI:** 10.3390/s20030817

**Published:** 2020-02-03

**Authors:** Dan Popescu, Florin Stoican, Grigore Stamatescu, Loretta Ichim, Cristian Dragana

**Affiliations:** Faculty of Automatic Control and Computers, University Politehnica of Bucharest, 060042 Bucharest, Romania; florin.stoican@upb.ro (F.S.); grigore.stamatescu@upb.ro (G.S.); loretta.ichim@upb.ro (L.I.); cristian.dragana@gmail.com (C.D.)

**Keywords:** unmanned aerial vehicles, wireless sensor networks, intelligent data processing, trajectory planning, relevant data extraction, data consensus, Internet of Things, precision agriculture

## Abstract

The growing need for food worldwide requires the development of a high-performance, high-productivity, and sustainable agriculture, which implies the introduction of new technologies into monitoring activities related to control and decision-making. In this regard, this paper presents a hierarchical structure based on the collaboration between unmanned aerial vehicles (UAVs) and federated wireless sensor networks (WSNs) for crop monitoring in precision agriculture. The integration of UAVs with intelligent, ground WSNs, and IoT proved to be a robust and efficient solution for data collection, control, analysis, and decisions in such specialized applications. Key advantages lay in online data collection and relaying to a central monitoring point, while effectively managing network load and latency through optimized UAV trajectories and in situ data processing. Two important aspects of the collaboration were considered: designing the UAV trajectories for efficient data collection and implementing effective data processing algorithms (consensus and symbolic aggregate approximation) at the network level for the transmission of the relevant data. The experiments were carried out at a Romanian research institute where different crops and methods are developed. The results demonstrate that the collaborative UAV–WSN–IoT approach increases the performances in both precision agriculture and ecological agriculture.

## 1. Introduction

The need for high-performance, high-productivity, and sustainable agriculture results from the rapid growth of the human population. This requires permanent monitoring and intelligent processing of the measured data collected from the field, correlated with the weather forecasts, to produce agronomic recommendations. In the last few years, new technologies in agriculture, and especially in precision agriculture (PA), have been leveraged for increased productivity and efficient input dosage [1]. Most importantly, in PA, farmers need to know exact and timely details about crop status. These details about certain parameters, obtained by measurements both from the ground and in the air, constitute input data to specialized systems of process management in the PA. Some relevant examples might include for example, irrigation control, pesticide dosage, pest control, etc. For acquisition and complex processing of the collected data, integration of unmanned aerial vehicles (UAV) with wireless sensor networks (WSN) under novel frameworks, such as the Internet of Things (IoT), has been shown to contribute to increases in agricultural yields [2]. Such advanced systems are modeled as well-specified agent-based solutions with sensors and UAVs. Although the contributions of UAVs and WSNs, taken separately, are well documented and important in the sustainable growth of agricultural production, the integration of these components together within an IoT framework is expected to significantly improve the solutions for monitoring, production modeling, prediction, and decision-making.

Relevant applications of UAV–WSN systems are presented in Reference [3,4,5,6]. In viticulture, as a special type of PA, the soil and air parameters modify grape yield and quality. For this purpose, a solution based on the collaborative system mini UAV (quadrotor type)–WSNs to monitor parameters, like temperature and humidity, to prevent the frost in fragmented vineyards is proposed in Reference [3]. The UAV is considered as communication relay between sensors and a base station. A real application for monitoring sensitive parameters in vineyards with both agro-meteorological stations and UAV platforms is presented in Reference [4]. In order to obtain a precise monitoring of the specific indicators, the data from the ground are correlated with the data collected by a UAV platform with 8 rotors provided with a professional thermal camera. The study was conducted over a period of two years. For data acquisition on large areas, a fixed wing type UAV, used as data mule from the ground WSN, is proposed in Reference [5]. In addition, the UAV has attached an high definition (HD) camera for the detection of certain plant diseases. Experimentally, a small tank has been added to spray different insecticides, fertilizers, herbicides, etc. Both UAV and WSN are low cost and not robust for only demonstration purposes. In addition, in Reference [6], a low cost agro-meteorological monitoring system in a vineyard was designed and developed. The optimal positioning of the sensors was made with the help of the multispectral image analysis, acquired by UAV.

Given recent evolutions in UAV technologies, cost reduction, and new regulations of aviation authorities regarding the usage and deployment of such systems (e.g., European Aviation Safety Agency (EASA) [7] and Federal Aviation Administration (FAA) [8]), such aerial robotic platforms are increasingly used in agriculture for different tasks, the most important being crop monitoring [9]. According to EASA, the UAVs should be safely integrated into the existing aviation context in a proportionate way [7]. For large scale applications, in which UAVs are flying beyond line-of-sight, compliance with strict regulatory frameworks is essential.

Adoption of a UAV-based solution for image acquisition in agriculture applications is more cost effective and flexible in comparison with satellite or manned aircraft alternatives [10]. Both fixed- wing [11,12] and rotary-wing type [3,13] UAVs are frequently used in various applications in agriculture, while accounting for the risk of crashes [9] and potential damages. Equipped with specific sensors in modular payloads [14], such as high resolution RGB [15], infrared, multispectral [16,17], thermal cameras [18,19], and also LIDAR [10], UAVs are able to create precise maps of crop state or evolution [17], health plant assessment [20], diseases [21], soil characteristics, evaluate losses caused by floods [11], etc. In the crop monitoring, the following characteristics are analyzed from UAV images [9]: the crop water stress, defined as the difference between the canopy and the air temperature, the photochemical reflectance index, and the vegetation indices.

Although UAVs with different propulsion systems are now available, most applications in PA use UAVs driven by electric motors due to their compact size, reduced maintenance and operational costs and, not the least, their alignment with the current regulatory context and tendencies towards the reduction of global carbon emissions [22].

The small-scale data acquisition by the WSN helps farmers to take actions like crop irrigation, fertilizer usages, deciding on the optimum stages of sowing, and harvesting [23]. Moreover, WSNs employed in PA lead to large amounts of data. Thus, data collection by WSNs is an important contribution to the development of farm management information systems (FMIS) [24,25].

The WSN has multiple functions at the field level: data acquisition of various parameters (e.g., temperature in soil and air, humidity in soil and air, solar radiance, soil nutrients, the presence of pests and weeds, chlorophyll content in plants, etc.), distributed processing of data by establishing consensus—if it is the case, establishing the relevant data and its storage, low level data fusion, and data transmission. New sensor node designs offer reduced costs [26]; see, for example, the detailed list of sensors used in PA given in Reference [10]. As in many other large-area monitoring applications, for communication or local processing reasons, the sensors are grouped into sensor networks, the communication being made by radio. A WSN network will include measurement nodes (sensory nodes) and data collection, processing, and transmission nodes (sink or cluster head).

Regarding PA, there is no rigorous theory of sensor placement because it depends on the particularities of the soil and the weather. Sensor groups need to comply broadly with the need for sensory and communication coverage. In Reference [27], two examples of sensor location topologies are given: grid and random. From the point of view of communication with the sink node, the most used are the star and mesh topologies. The wireless communication protocols used in WSN for PA are the following [10]: 6LoWPAN, ZigBee (both being the most suitable for the mesh topology), LoRaWAN, GSM, BLE, and Wi-Fi.

In PA, WSNs are used, most often, for parameter monitoring, but they can also be integrated into control systems as sensors. Direct specific applications of WSN in control systems for PA are the energy efficient automated control of irrigation [28] and smart automated fertilization [29].

The performance of the crop monitoring can be improved by UAV–WSN collaboration [30]. The collaborative aspects in an integrated UAV (aerial agents)–WSN (ground agents) architecture for different applications was recently presented in a review paper [22], where the different functional components of the system and how they collaborate with each other was highlighted. In Reference [31], the authors presented an integrated UAV–WSN–IoT system, named FarmBeats, which is an end-to-end platform for data collection from various sensors, cameras, and drones in agricultural applications. An unlicensed TV White Spaces is used to setup a high bandwidth link from the farmer’s home to an IoT ground station at a distance for collecting data from UAVs and WSNs.

In order to interconnect the UAVs and terrestrial WSNs into hybrid networks and, at the same time, to ensure a safe airspace sharing with aircrafts, multiple organizations are contributing [22]: International Civil Aviation Organization, EASA, Joint Authorities for Rulemaking on Unmanned Systems, International Telecommunications Union, etc. Satellite connection is required for two reasons. One-way communication, such as obtaining the GPS location of the UAVs or the sensory nodes (if any) is one reason. The second reason is a possible data transmission or remote control (via two-way satellite-intermediated internet).

In Reference [32], the authors discuss the information system design supporting agriculture data management. Enabling advanced data processing in the form of sensor fusion and clustering mechanisms for improved network topologies in generic applications has been discussed [30]. Effective data gathering mechanisms [33] and higher level IoT architectures [34] are key and current topics of interest.

We believe that the challenges of UAV–WSN–IoT integrated systems can come from several directions: (a) precise localization of the ground sensors with the aid of a preliminary flight; (b) sensor states periodically inspected by UAV; (c) establishing of the WSNs as sensor clusters able to cover, both from the sensorial and from the communication point of view the monitored area; (d) establishing the cluster heads (CH), named base stations, of the WSNs able to communicate data to UAVs; (e) transmitting commands to change the strategy and parameters of the sensor networks, (f) data acquisition from WSNs through UAVs, (g) special trajectory planning and tracking, (h) the aggregation of information collected by the UAV with the information collected by WSN for the purpose of measuring and interpreting the parameters with increased accuracy, (i) remote control via Internet, and (j) edge and cloud computing.

In a hierarchical structure, the data processing architecture of the integrated system is based on three levels: consensus, edge computing [35], and cloud computing.

For the main activity, the data collection from CH, UAV must have a predefined trajectory, properly designed, and accounting for the following limitations:

- Waypoint passing: a UAV has to pass above the CH to extract the relevant data from that area (covered by the corresponding WSN sub-network);
- Obstacle avoidance: UAVs avoid obstructions or prohibited areas along the flight plan;
- Guaranteed communication: to ensure that the data has been fully collected, enough time has to be spent in the CH neighborhood;
- Efficiency: reduce at a minimum the energy consumption for that trajectory (consider the length of the trajectory and its complexity).

The integration of UAV–WSN based systems for PA in IoT is a mandatory step to create an advanced FMIS [25].

Due to the integration, the system can become “smart” by using elements of artificial intelligence like self-adaptation and decision, optimal trajectory, data transmission of relevant parameter values, energy efficiency, and neural networks for data and image processing. Not in the least, the sensors must be placed optimally, considering the terrain characteristics. Battery life is an important design point of the ground sensor algorithms by reducing to a minimum the number of wireless communications needed to transfer the information. The radio interface is the critical factor in increasing battery life. Based on the frequency of the data collection and radio transmissions the nodes can have a battery lifetime ranging from several months up to one year. Therefore, the intelligent collaboration between UAV and WSN can lead to optimization of parameters, such as energy consumption, sensing coverage, risk, data acquisition, and processing time [36]. To this end, bio-inspired optimization heuristics and genetic algorithms were applied to the aforementioned agents.

The optimal WSN coverage by the aid of UAV platforms is implemented in Reference [37] as an optimization problem, formulated by means of the travelling salesman problem, in order to find the best path of the UAV for data collection with minimum energy consumption.

Using UAV as data mule for multi WSNs is an energy-efficient method to increase the networks’ life. To this end, the authors in Reference [38] apply the successive convex optimization technique.

The proposed system presents the following integration aspects:

- Group the sensors in clusters and determine the cluster heads, the methodology proposed by the authors in Reference [30];
- Path planning based on specific conditions for efficient data collection; and
- Intelligent data collection and processing.

The main contributions consist in the following: (i) implementation of a multilevel, collaborative UAV–WSN system structure for agriculture applications, (ii) a specific path planning for fixed wing–type UAV with the purpose of robust and efficient data collection, (iii) obtaining relevant data from sensors for the purpose of saving energy, and (iv) edge–fog–cloud computing algorithms for subsequent data processing. Thus, the main challenge is related to improving data extraction and communication in large scale heterogeneous monitoring system. The key problem is focused on improving the performance of such systems through better algorithms and synchronization among the two subsystems: the ground sensor network and the robotic aerial platforms, implemented as UAVs, for data collection and relaying.

The rest of the paper is structured as follows. Section 2 describes the concept, the methodology, and key aspects that have been addressed for the proper design and implementation of the system. Section 3 presents the experimental results and performances after implementing the system on an experimental farm. Section 4 highlights the conclusions, as well as future work.

## 2. Materials and Methods

### 2.1. Requirements for Integrated UAV-WSN-IoT Systems

**Figure 1:**
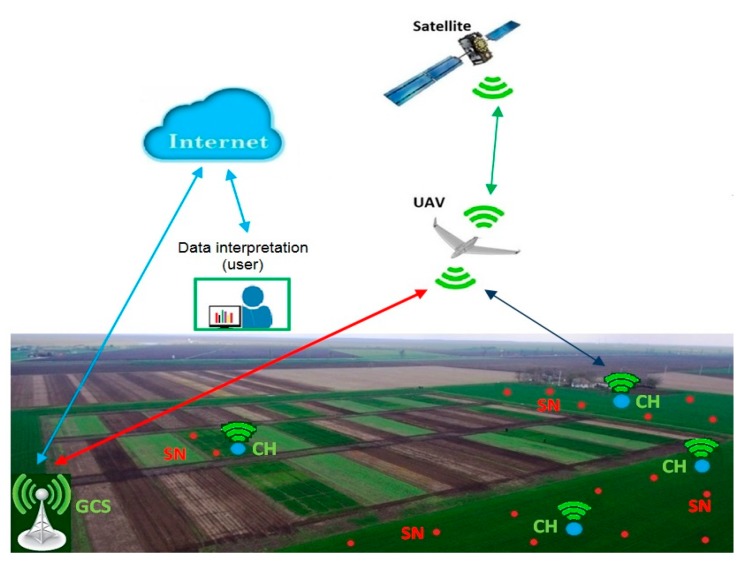
The concept of the integrated unmanned aerial vehicle (UAV)–wireless sensor network (WSN)–Internet of Things (IoT) system.

For the design of reliable and robust large-scale monitoring system the requirements have to first be validated. The main challenges for such collaborative systems were considered to be: sensing coverage in accordance to mission objectives, communication coverage by the hybrid UAV–WSN system using various types of radio links, from low-power, low-data rate to high throughput long distance for streaming, energy efficiency, and, not in the least, computing efficiency. The decentralized architecture for crop field monitoring described in this paper is designed to overcome the challenges mentioned above and to account for the data generation patterns at the field level. While the proposed data fusion mechanisms and processing of centralized in-field data at CH level manage to reduce data volume and ensure the flow of information up to the level of events, an additional intermediate level is appended to the data stream, in order to reach the server. To this end, we consider both mobile agents (UAV) and multiple fixed agents (ground sensors (SNs)). The system diagram is presented in Figure 1. The mobile agent can perform the following functions: data mulling, image acquisition, relay, and state inspection of WSNs. The fixed agents acquire data from the field (agricultural field—soil and air), process data locally (relevant data extraction, data consensus), and finally transmit data to the UAV by means of CHs. The system is composed of four main processing levels (Table 1): Sensor level, Fog Computing level, Internet/Cloud Computing level, and Data Management and Interpretation level. This is a multi-WSN–UAV structure with higher level integration in internet-based systems for decision support. The data from WSNs are collected by a UAV, transmitted at a ground control station (GCS), and, from here via the internet, to the Data Interpretation module. Analytics functionality ranges from basic statistical indicators to trend and event detectors and up to basic statistical learning models that have the ability to anticipate evolutions in the monitored ground phenomena.

Another important requirement of the integrated system is the correlated or complementary interpretation of the data from the sensory agents, either mobile or fixed. For example, when the soil moisture is too high, the soil sensors show the maximum value and cannot discern whether a flood has occurred. This can be accurately determined from aerial images taken by the UAV. In addition, the degree of humidity in plants and the degree of foliage development can be observed either from the ground or from the air (images), and a more precise determination results from the fusion of the two data sets.

Other types of similar systems were surveyed and can include the use of swarms of multi-copter type UAVs, which offer better positioning accuracy for data collection while trading off energy efficiency and autonomy. Ground sensor network implementation can also be a differentiating factor with two main approaches: random deployment of sensor nodes in the area of interest, according to a minimum expected sensing coverage density, or deterministic, grid-like placement. Intermediate data processing steps from the field level to the decision level are commonly accepted as an important mechanism to balance network loads and improve communication latency.

### 2.2. UAV Trajectory Design

For UAV trajectory planning, two cases must be considered. The first is the trajectory planning for collecting data from sensors (CHs), and it must take into account certain requirements, such as the complete and safe acquisition of data, on one hand, and minimize energy and time consumption, on the other hand [39].

Under certain reasonable assumptions (known environment, known limitations), the UAV tasks reduce to computing a trajectory which respects constraints and minimizes a cost (length, total energy consumption, etc.), while simultaneously respecting various constraints (internal dynamics, stall velocity constraints or exogenous ones, those imposed by the environment, such as obstacle avoidance and waypoint passing through).

The particularity lies in the fact that many of the UAV-specific constraints are non-convex [40], e.g., the variable of interest *z* (depending of time *t*) has to stay outside some bound (e.g., outside of an interdicted region and/or maintain a minimal velocity). If *z*(*t*) is the UAV position, the velocity restrictions are usually written as follows:

(1) $$\underline{v}\leq \left|\left|\dot{z}\left(t\right)\right|\right|\leq \bar{v}.$$

Both bounds (lower—$\underline{v}$ and upper—$\bar{v}$) may depend on a variety of factors. Hard constraints are imposed by the UAV physics: upper bound given by the engine characteristics and lower bound by the requirement to avoid stall. Note that this work neglects the influence of wind: velocity is usually measured against the ground (e.g., through a GPS), but, in fact, the UAV “feels” the addition of its own and of the wind velocities. This may lead to an unexpected stall or, at least, improper behavior. Usual techniques are to provide more conservative bounds in Equation (1) and to restrict the flight to normal weather conditions.

**Figure 2:**
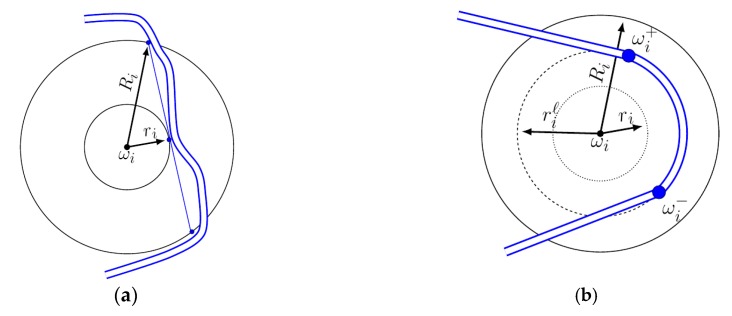
Illustration of different aspects of the trajectory design: (**a**) inner and outer communication constraints with a sufficient condition and a corridor for the UAV trajectory envelope and (**b**) trajectory validating.

Waypoints are introduced, in a practical mission, because data has to be gathered from a cluster node. Thus, the question of minimum communication time arises [41]: It is necessary to remain in a specific neighborhood for a defined time interval Δ*t_i_*. To correctly describe such a constraint, we require a tuple $\left(\omega_{i},\Delta t_{i},r_{i}\;,R_{i}\right)$, where $\omega_{i}$ is the corresponding cluster node position (the center of the circle in Figure 2), and *r*_i_ and *R*_i_ are, respectively, the minimum and the maximum radius of the permitted communication area. Because there are perturbations due to trajectory control errors or other causes, the real trajectory is included in a flight lane (Figure 2a). The flight lane was experimentally established at 30 m, under reasonable assumptions about wind speed. The trajectory z(t) has to stay near the waypoint for a least amount of time $\Delta t_{i}$ determined by the quantity of data which has to be transferred:

(2) $$r_{i}\leq \left|\left|\omega_{i}-z\left(t\right)\right|\right|\leq R_{i},\;t\in \left[t_{i},t_{i}+\Delta t_{i}\right].$$

condition (2) is often impractical to check due to the continuous nature of z(t) and because of the varying time interval $\left[t_{i},t_{i}+\Delta t_{i}\right]$. The usual approach is to sample the constraint and to estimate the path length by assuming the bounds (1) on the velocity. To this end, we consider:

(3) $$\left|\left|z\left({\bar{t}}_{i}\right)-\omega_{i}\right|\right|=r_{i},$$

with ${\bar{t}}_{i}$ given such that ${\bar{t}}_{i}\in \left[t_{i},t_{i}+\Delta t_{i}\right])$ holds; it is important that a waypoint is reached, not when.

Note that the shortest distance for a trajectory checking (4) is the straight line shown in Figure 2a, whose length is $2\sqrt{R_{i}^{2}-r_{i}^{2}}$. In other words, a sufficient condition for guaranteeing that the minimal time $\Delta t_{i}$ has passed is to ensure that

(4) $$\Delta t_{i}\geq \frac{2\sqrt{R_{i}^{2}-r_{i}^{2}}}{\bar{v}}.$$

Condition (4) provides a lower bound for the time the UAV stays between the inner and outer circles (i.e., how much time it spends inside waypoint’s $\omega_{i}$ communication range). Then, inserting (3) in a trajectory design procedure implicitly guarantees enough communication time. This approach may be insufficient for a couple of reasons. First, the desired communication time may not be known at the trajectory generation time and thus could not be compared with $\Delta t_{i}$. Second, the communication time is known to be larger than $\Delta t_{i}$ and a “tangential” pass (like the one enforced by (3)) does not suffice. The method (detailed below) is to enter a loitering mode to increase arbitrarily the data-gathering time [42]. Making the reasonable assumption that the loitering $r_{i}^{l}$ radius respects the condition $r_{i}<r_{i}^{l}<R_{i}$, means that the UAV can orbit the waypoint $\omega_{i}$ for an indefinite period of time [43]. From the viewpoint of trajectory generation, the only relevant question remains the places at which the UAV inserts/dislodges onto/from the loitering circle. Both of these points are decided by the relative position of the current waypoint with respect to the previous and next waypoints in the sequence (such as to reduce unnecessary inflexions in the trajectory). The switch between normal and loitering modes will be done at pre-determined points: the trajectory enters loitering mode at a point $\omega_{i}^{-}$ and dislodges from it at a point $\omega_{i}^{+}$ (which lie on the loitering circle and are from/towards the direction of the previous/next waypoint). Thus, when the UAV decides to finish the communication, it will continue to orbit the loitering circle until it reaches the dislodging point $\omega_{i}^{+}$. Here, it will switch back to the normal trajectory mode.

The inner (dotted line), outer (solid line) communication circles, and loitering circle (dashed line) are illustrated in Figure 2b. We show a trajectory inserting to the loitering circle, tracking an arc of it, and, lastly, dislodging from the circle to re-enter its normal mode (line tracking). The UAV could have orbited the loitering circle repeatedly and dislodged from it at $\omega_{i}^{+}$ when desired. As is mentioned above, the trajectory describes a corridor (we account for the inherent tracking error appearing under realistic conditions).

While the previous velocity and time constraints are easy to formulate, they lead to complex (nonlinear in position and time variables) constraints. Thus, in practical implementations, it is often much easier to provide a simplified control scheme based on the heading angle (a “line of sight” procedure).

That is, the UAV control is partitioned into the lower level where the velocity is controlled (to negate the wind disturbances, for example) and the higher level where, at each time instant, a new heading angle is computed. Thus, we may interpret the path as a collection of segments (linking consecutive waypoints) and circle arcs around waypoints where loitering is needed.

**Figure 3:**
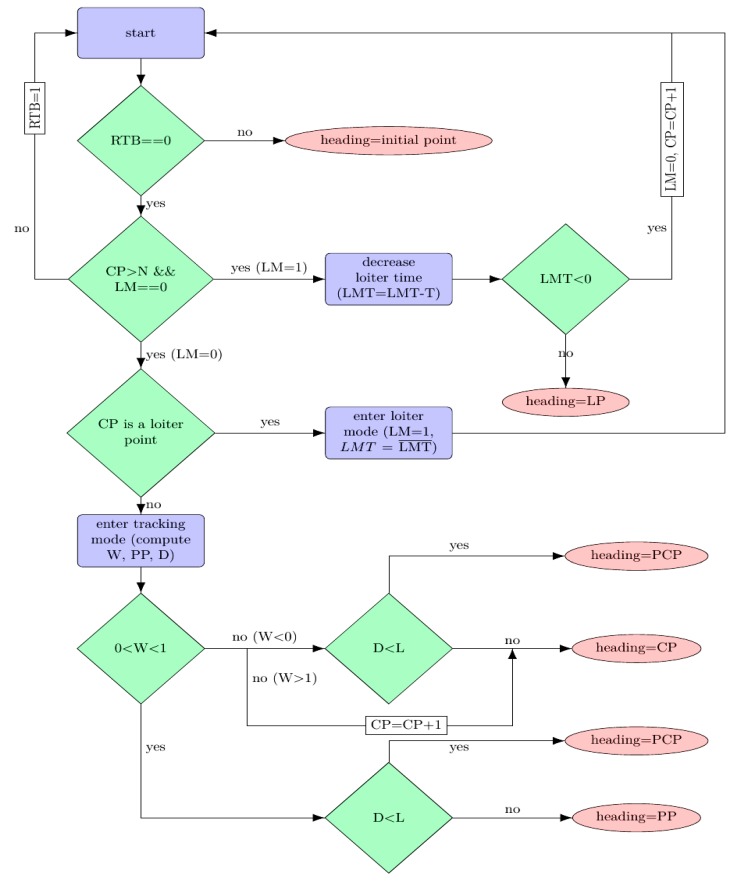
Flowchart for the path planning. RTB = return to base; CP = current waypoint; LM = loiter mode; LMT = loiter mode remaining time; T = constant value; LP = loiter point; PP = projection point; W = weight of the PP; D = distance between the UAV position and the PP; PCP = proximity circle point; L = vector length.

The idea of the segment tracking procedure is straightforward and is sketched in the following flowchart (Figure 3). In the flowchart, we make use of several notations:

- RTB = return to base, a flag denoting whether the UAV has to return to its path’s starting point;
- LM = loiter mode, denotes that the UAV has entered the loiter mode; at the start of this mode, the LMT = loiter mode remaining time is initialized to a predefined value which is decreased (at each step with a constant value T) as long as the UAV remains in the loiter mode;
- PP = projection point, obtained by projecting the current position onto the support line of the current segment from which W = weight of the PP (denoting whether the PP is inside the segment, to the left or to the right) and D = distance between the UAV position and the PP, are computed;
- PCP = proximity circle point represents the intersection between the proximity circle and the current segment (in case of intersection between the circle and the segment there are two solutions; the one closest to the end-point of the segment is taken);
- LP = loiter point is computed such that the UAV tracks the loiter circle (with the sense of movement decided a priori by the supervisor); and
- CP = current waypoint, throughout the algorithm, is updated as needed.

The main points of the algorithm are:

► The UAV has two modes of functioning, loiter mode and segment tracking mode, which are decided by the supervisor (in the sense that within the collection of waypoints a priori computed, some of them are labeled as loiter points).
► In both cases, the algorithm provides a heading which is the reference to be tracked by the UAV. This is in line with standard practices, where the heading is decided through some design procedure and the velocity and pitch and roll angles are decided at the auto-pilot level (usually the velocity is maintained constant and the roll and pitch are taken as needed between admissible bounds).
► The decisions taken by the algorithm and supervisor are, ultimately, related to the distance between the current position and some point of interest. To do so, we consider some circles of interest, defined as follows:
  ○ Communication circle: the UAV communicates with the ground-based cluster head only when it is within the communication radius.
  ○ Waypoint update circle: it is impractical to assume that the UAV passes through the exact coordinates of the current waypoint. Thus, we update the active segment (by advancing through the list of waypoints) whenever we are close enough to the end-point of the current segment.
  ○ Loitering circle: whenever the UAV is required to spend a significant time in communication with the current cluster head, the decision to start loitering is taken. The loitering radius is restricted to be less than the communication radius and larger than the physical limitations imposed by the roll angle bounds (a tighter circle means a larger roll angle).
  ○ Proximity circle: the procedure employed in the algorithm takes (whenever there is intersection between the circle and the current segment) the heading angle in the direction of the intersection point (the one closest to the end-point of the segment).
► When the last waypoint is covered, the UAV returns to base (by default, we consider this to be the initial point on the trajectory).

Without being exhaustive, some of the most relevant updates in the algorithm are:

In segment tracking mode:

1. At the current time, we consider the UAV position (*x*,*y*), the segment determined by the current (CP) and next waypoint (CP + 1): $\left(w_{x}^{i},w_{y}^{i}\right),\;\left(w_{x}^{i+1},w_{y}^{i+1}\right).$
2. We compute the projection of the current point onto the current segment (PP). We identify three possible cases by checking the relative position of the projection wrt the segment’s end points (described by *W*): inside the segment (0 ≤ *W* ≤ 1)), outside and located before the initial segment end (*W* < 0); outside and located after the initial segment end (*W* > 1);
3. We compute the distance (D) from the current point to the segment and the circle of radius *L* (proportional with the UAV velocity) and further used to compute the heading vector.
4. We consider the following cases:
  - The UAV is too far away, and the projection point lies before the segment start point. Then, the heading angle points towards the projection point.
  - The UAV is sufficiently close, and the projection point lies before the segment start point. Then, the heading angle points towards the start point.
  - The UAV is sufficiently close to the segment end point, or its projection onto the segment lies after the end point. Then, the current segment is updated, and the procedure jumps to step 4i.
  - The UAV is too far away, and its projection lies onto the interior of the segment. Then, the heading vector points towards the projection.
  - The UAV is sufficiently close, and its projection lies onto the interior of the segment. The heading angle is taken as the vector of length L in which the tip lies on the segment (there are two possible tips; the one closer to the segment end point is considered).
5. Go to step 1.

In the loitering mode:

1. Select the loitering center as the current waypoint.
2. Construct the circle of radius L and centered in the current position of the UAV.
3. If the circle does not intersect the loitering circle, move towards the projection point situated on the loitering circle.
4. If the proximity circle intersects the loitering circle, take the heading vector along the tangent at the intersection point between loitering circle and proximity circle (there are two solutions, we selected depending on the desired loitering rotation—clockwise or counterclockwise).

Note that all steps where a decision regarding the trajectory update is taken consist in fact in a decision about the UAV’s heading. Thus, for trajectory tracking, only the heading angle is used as control input. This suffices for relatively simple trajectories and is robust against wind disturbances (as later shown in the simulations).

### 2.3. Relevant Data Extraction

The collected data is hierarchically processed from the ground level, cluster head level, UAV level up to the cloud. Alongside these steps, information is gradually extracted through various methods that enable local decisions based on the configuration of the system (thresholding, consensus, symbolic aggregate approximation, etc.).

**Figure 4:**
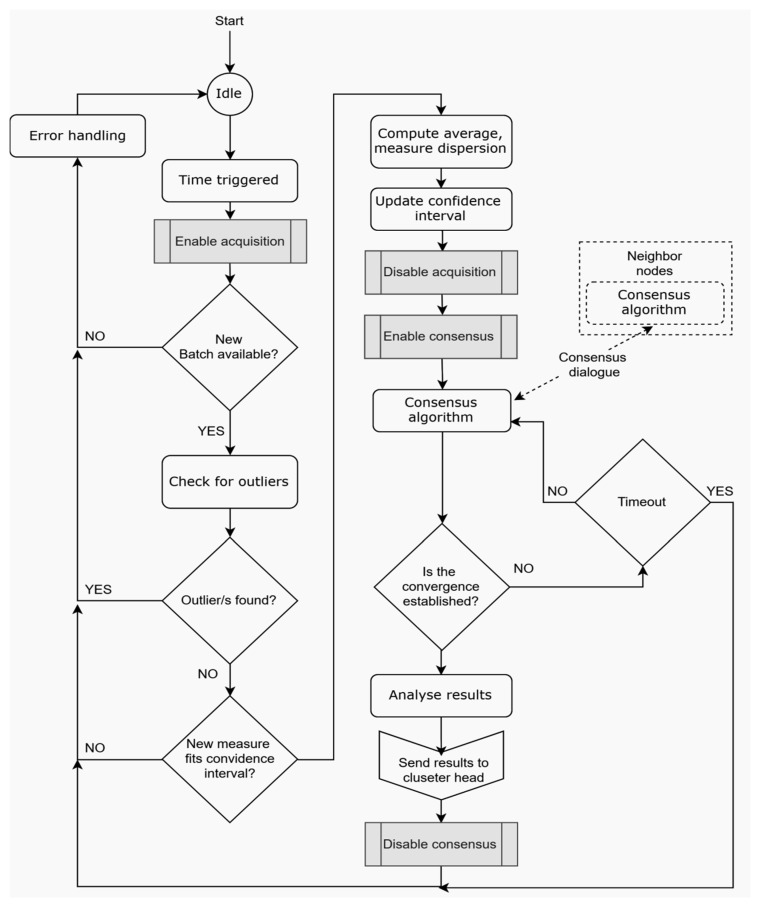
Flow diagram of the data processing steps at the field level, based on consensus algorithm.

In-field data processing is ensured both at local level (independent data filtering) and decentralized at network level (through data exchange between neighbor sensory nodes). The proposed data processing mechanisms, tailored for in-field level, are designed in order to ensure a substantial weighted average. This step is found as ‘Enable consensus dialog’. Once the convergence is reached, each node performs a routine for results analysis basically seeking to discover and mark nodes with divergent values. This information remains available alongside the consensus value so that it can be interrogated by the higher level of data processing if needed. This is found in Figure 4 as ‘Analyze results step’.

Aggregated data sets are achieved through different methods. All seek for relevant data points, aiming to a reduced size set and providing at the same time a satisfying reconstruction of the initial data. The proposed method for data aggregation is based on the minimum and maximum values extraction, computed as global extremes for a predefined period of time (e.g., a day). It is obvious that this method is suitable only for measurements that have a periodic behavior, with smooth variations during the day. A measurement for which this method is suitable is the soil temperature. Conversely, change detection is commonly used for irregularly-shaped data sets. This method follows extraction of local extreme points where trend changes occur.

Given a set of data points $\left(x_{i},\;\;y_{i}\right),\;i=1,\;\ldots ,\;n$, trend $t_{i}$ is computed for each sequence measurements such that for a measure m, (5),(6),(7) has to be true. If $t_{i}\neq t_{i+1}$, then it means that a trend change has occurred, and the data point $\left(x_{i},\;y_{i}\right)$ is added to the relevant data set.

(5) $$x_{i+1}^{m}-x_{i}^{m}>\delta^{m}\Rightarrow \;t_{i}^{m}=1,\;$$

(6) $$x_{i+1}^{m}-x_{i}^{m}<-\delta^{m}\Rightarrow \;t_{i}^{m}=-1,\;$$

(7) $$x_{i+1}^{m}-x_{i}^{m}\in [-\delta^{m},\delta^{m}]\Rightarrow \;t_{i}^{m}=0.\;$$

Data collection is done periodically, following a succession of specific routines. As mentioned before, the first step for in-field data processing is performed at the local level, independently, by each sensor node.

**Figure 5:**
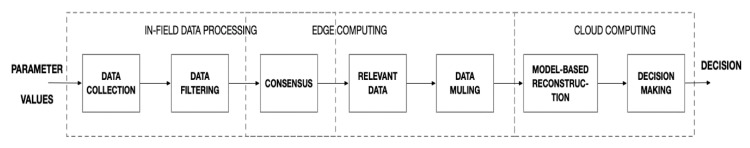
Flow diagram of the data processing at the system level.

While the proposed data fusion mechanisms and processing of centralized in-field data at gateway level manage to reduce data volume and ensure the flow of information up to the level of events, an additional intermediate level is appended on the data stream, in order to reach the server. Consequently, the system is composed from three processing levels (Figure 5): In-field data processing, Edge computing, and Cloud computing. This corresponds to a UAV–WSN system with internet integration. The data from WSNs are collected by a UAV (or team of UAVs) and then transmitted at a ground control station (GCS). From here, the data is transmitted, via the internet, to the Cloud computing level and, finally, to the ‘Data interpretation and decision’ module.

In a consensus mechanism, multiple autonomous agents seek to reach the convergence value under the influence of the information flow exchanged inside the network. Each node updates its estimated value using an updating rule. An update law for node $n_{i}$ based on local weighted consensus is described by the following equation:

(8) $$x_{i}\left(k+1\right)=\omega_{ii}x_{i}\left(k\right)+\sum_{j\in N_{i}}\omega_{ij}x_{j}\left(k\right),$$

(9) $$\sum_{i\in M}^{}\sum_{j\in N_{i}}^{}\omega_{ij}=1,$$

where

$x_{i}\in \;\mathbb{R}$ is the computed estimate of node i;
$\omega_{ii}$ is the weight applied to its own previous computed estimate;
$\omega_{ij}$ is the weight associated with the node j for the value of node i;
k is a convergence step; and
$N_{i}$ is the neighborhood of node i, i∈{1, 2, 3, …, m}=M.

The proposed consensus algorithm is built using a hybrid weighted average consensus which ensures that the updating rule computes the current convergence value, keeping a high priority for the closest neighbors, but at the same time, it aims at suppressing outlier values.

Each node computes the weights $\omega_{ij}$ based on the distance $d_{ij}$ computed using the available location information.

(10) $$\omega_{ij}=\;\{\begin{array}{ll} \frac{d_{min}}{d_{ij}} & if\;\left(i,j\right)\in \varepsilon ,\;i\neq j\\ 0 & if\;\left(i,j\right)\notin \varepsilon ,\;i\neq j \end{array},$$

where

$d_{min}$ is the distance to the closest neighbor; and
$d_{ij}$ denotes the distance between node i and j.

Using the selected weights, the algorithm performs a weighted average of neighbors values defined as:

(11) $$N_{i}mean\left(k+1\right)=\;\frac{\sum_{j\in N_{i}}\omega_{ij}x_{j}\left(k\right)}{dim(N_{i})}.\;$$

In order to suppress outlier values, additional weights are applied for previously computed estimate $x_{i}\left(k\right)$ and current neighborhood estimate average $N_{i}mean\left(k+1\right)$. Thus, this is an auto-supressing mechanism computed as the ratio between the standard deviation at convergence step k+1 and the deviation of the previous estimate $x_{i}\left(k\right)$. This is written as:

(12) $$\begin{array}{c} x_{i}\left(k+1\right)=\;\frac{\Delta \left(k+1\right)\cdot x_{i}\left(k\right)+\delta \cdot N_{i}mean\left(k+1\right)}{\Delta \left(k+1\right)+\delta }\\ \Delta \left(k+1\right)=\frac{\sqrt{\frac{\sum_{j\in N_{i}}{\left[x_{j}\left(k\right)-N_{i}mean(k+1)\right]}^{2}}{N_{i}-1}}}{\sqrt{{\left[x_{i}\left(k\right)-N_{i}mean(k+1)\right]}^{2}}},\\ \delta =1-\;\Delta \left(k+1\right) \end{array}$$

where

- Δ(k+1) is the weight applied to the state value, computed for each step of the average consensus;
- δ is the weight applied to the neighborhood estimate.

Once the consensus is reached, each node performs a routine for results analysis basically seeking to discover and mark nodes with divergent values. This information remains available alongside the consensus value so that it can be interrogated by the higher level of data processing if needed. This global mechanism indicates problematic sensor nodes or even very isolated events, but it cannot discern between them.

The flow diagram presented in Figure 5 shows the data processing pipeline for the integrated UAV–WSN–IoT system. Based on preliminary parameterization, e.g., sample rate, coverage area, and energy aware communication, sensor measurements are collected at the ground level by the local nodes. On-board basic data filtering is carried out to check the consistency and validity of the measurements for early detection of sensor faults, misreading or outliers. At the local network level, based on the validated and filtered data, consensus-based agreement is performed by in-network data processing, which leads to a common value for each of the acquired parameters among all nodes in a cluster. The cluster head further operates on the data by extracting relevant information through edge computing mechanisms, and a model-based compressed representation is achieved, e.g., polynomial interpolation models or more advanced methods, such as SAX (Symbolic Aggregate Approximation). At the conclusion of the edge computing phase, the UAV is activated for collecting the compressed representations of the ground phenomena from the cluster head nodes. The trajectory of the UAV is optimized as previously discussed to ensure timely collection from all the cluster heads in a target area and transfer the data to a central unit for back-end cloud computing processing and decision. The cloud computing layer integrates the data reconstruction based on the model parameters as inputs to a decision-making process, which yields the final outcome and allows for closing the loop by acting on the ground environment, e.g., irrigation and input dosage signals for the precision agriculture application.

When it comes to processing a large volume of data, many high-level representations of time series have been proposed for data mining, including Fourier transforms, wavelets, and piecewise polynomial models [44]. A different approach that we consider is the SAX algorithm, proposed in Reference [45]. This is a flexible method that allows adjusting the ratio between data volume and data relevance to ensure a fair reconstruction of original trends, while ensuring high data reduction by transforming of a time series into text strings. In essence, the algorithm operates by assigning label symbols to segments of the time series, thus porting it in a unified lower dimension representation. The importance of SAX’ parameterization must be considered by defining the number of segments and the alphabet size.

Starting with a time series 𝑋 of length 𝑛, this is approximated into a vector $\bar{X}=\left({\bar{x}}_{1},\ldots ,{\bar{x}}_{M}\right)$ of any length 𝑀 ≤ 𝑛, with 𝑛 divisible by 𝑀. Each element of the vector $\bar{x_{i}}$ is calculated by:

(13) $$\bar{x_{i}}=\frac{M}{n}\sum_{j=\frac{n}{M\left(i-1\right)}+1}^{{\left(n/M\right)}_{i}}x_{j}.$$

## 3. Experimental Results

**Figure 6:**
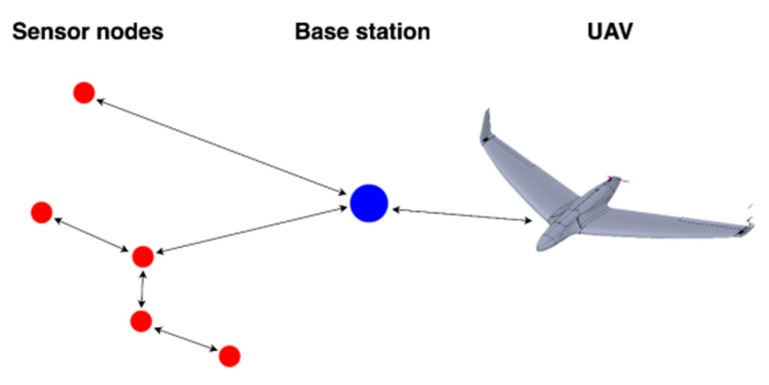
General configuration of UAV–WSN system implementation.

The high-level configuration of the integrated system is illustrated in Figure 6. The UAV is of the fixed wing-type, which enables coverage of large geographic areas with low energy consumption. The base station (CH) collects the primary data processed from the field sensors and periodically transmits it to a UAV according to its synchronization with the planned trajectory. Further, the data are processed in the cloud after the UAV uploads the collected data over the internet.

### 3.1. Path Tracking

**Figure 7:**
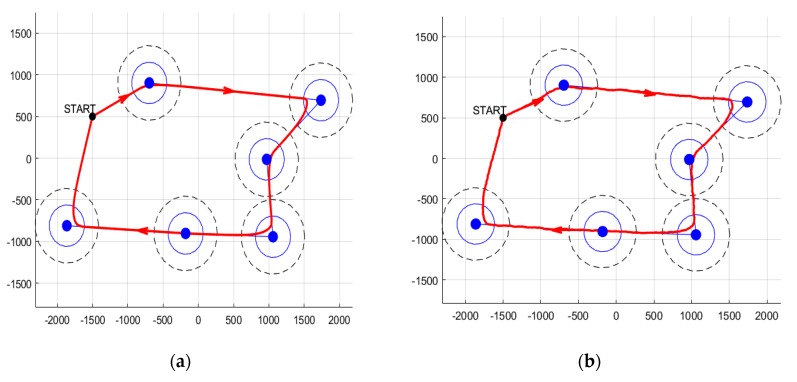
Illustration of segment tracking: (**a**) nominal case and (**b**) with wind disturbances.

We start by illustrating a nominal trajectory obtained by applying the segment tracking part of the path planning algorithm (Figure 7). The waypoints are the cluster heads (blue markers), and to each of them corresponds an update radius (solid blue line) and a communication radius (dashed black line). The first radius denotes the region in which an update of the current segment is carried out, and the second denotes the region inside which communication is possible. The starting point is chosen far away from the initial waypoint.

The algorithm provides, at each step, a heading vector which (with the use of the current position) leads to a heading angle. Together with a constant velocity value, these values are applied to a simplified 2 degrees of freedom UAV model, which is numerically integrated to provide the resultant path (solid red line). The sampling time is taken T = 1 s, and the numerical integration is done through ode45 in MATLAB 2018b.

The same scenario is carried out for the nominal case and for the case with wind disturbances (modeled by random uniform noise bounded by the interval [−15, 15]). The results are depicted in Figure 7, where we indeed observe a reasonable behavior of the resultant path (it passes through the waypoints neighborhoods, changes to a new segment as expected, and is smooth, at least in the nominal case).

**Figure 8:**
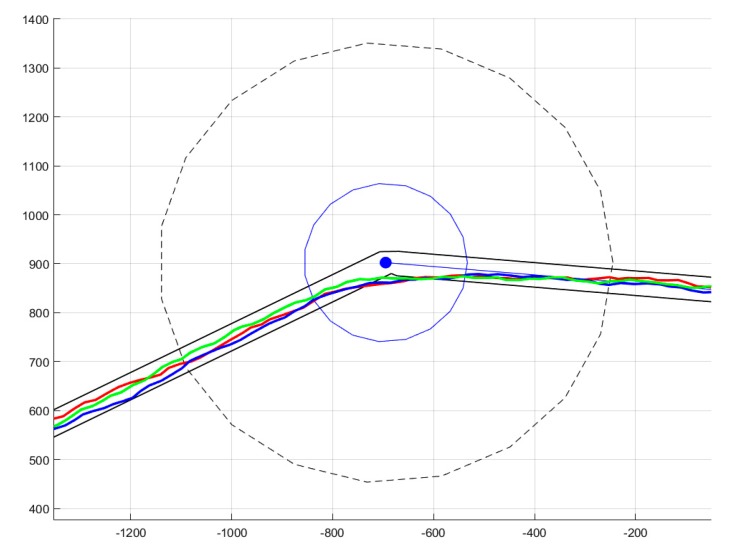
Illustration of trajectory tracking for multiple runs and with bounding corridor.

To better illustrate the scheme’s performance, we show multiple runs (3 samples), each of them for various noise values. We bound the resultant paths inside a corridor of diameter d = 30 m (Figure 8).

**Figure 9:**
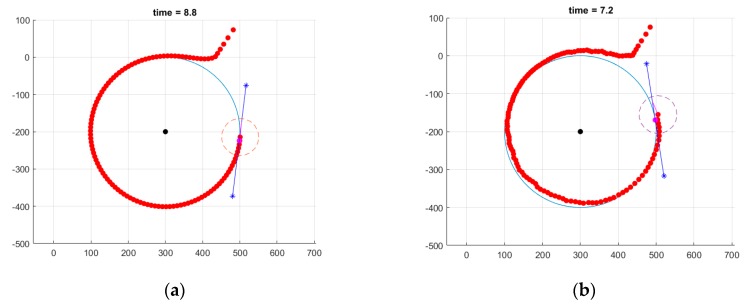
Illustration of loiter circle tracking: (**a**) nominal case and (**b**) with wind disturbances.

We observe that the resulted path does not guarantee enough time inside all communication ranges of the cluster head nodes. Specifically, we note that the 2nd and 6th waypoints (the one in the upper-most and the one in the lower-most corners) are only tangentially visited. Thus, the need for a loitering mode is clear. To better emphasize the behavior of the UAV when in loiter mode, we first show, in Figure 9, the path resulting in such a case (for both nominal and under disturbance functioning).

**Figure 10:**
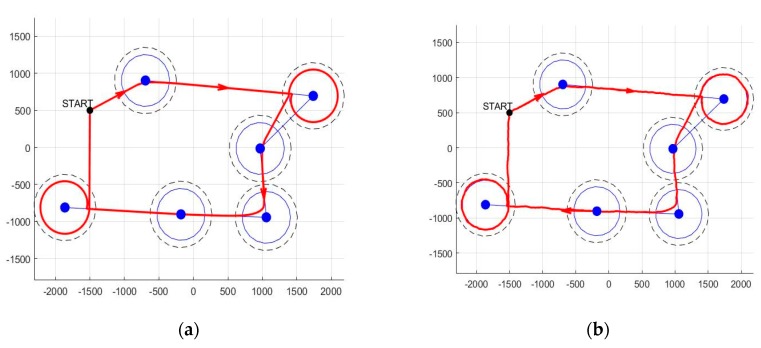
Illustration of combined (segment and loitering circle) tracking. In all cases, the loitering circle radius was taken to be 150 m: (**a**) nominal case and (**b**) with wind disturbances.

We can now integrate the full algorithm where we switch between segment and loiter modes, as needed. Specifically, in Figure 10, we consider that only waypoints 4 and 6 require the activation of the loitering mode and that the UAV stays in this mode for a fixed duration of t = 100 s. This can be obviously improved by deciding to exit the loitering mode at a later date (e.g., such that the UAV is already well-oriented towards the next waypoint).

**Figure 11:**
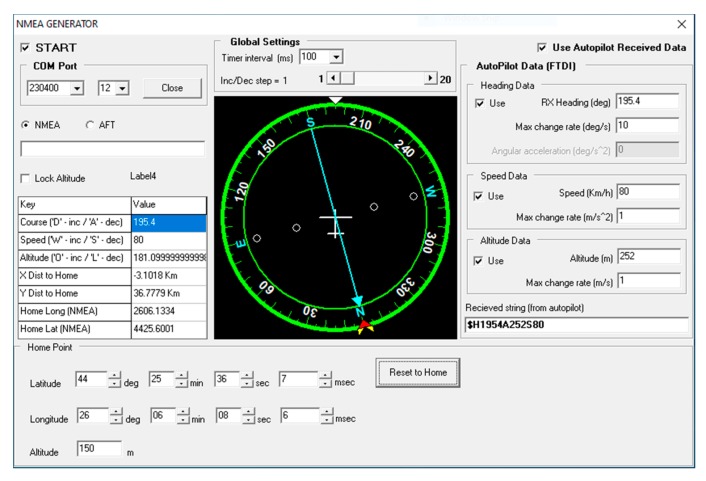
NMEA—based Simulator.

**Figure 12:**
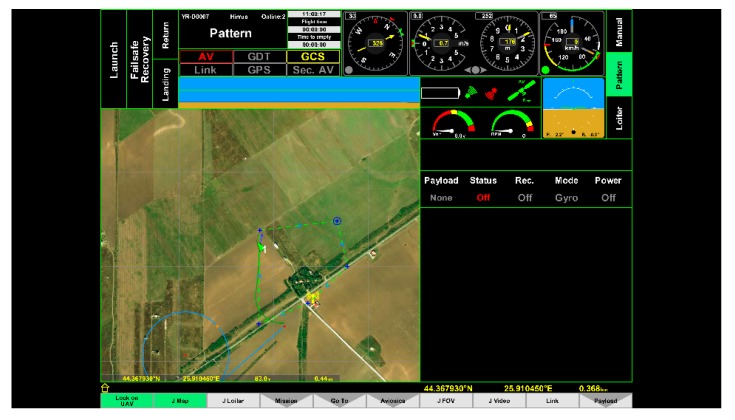
Pattern mode (tracking segments = green dashed line). Green arrow = UAV.

**Figure 13:**
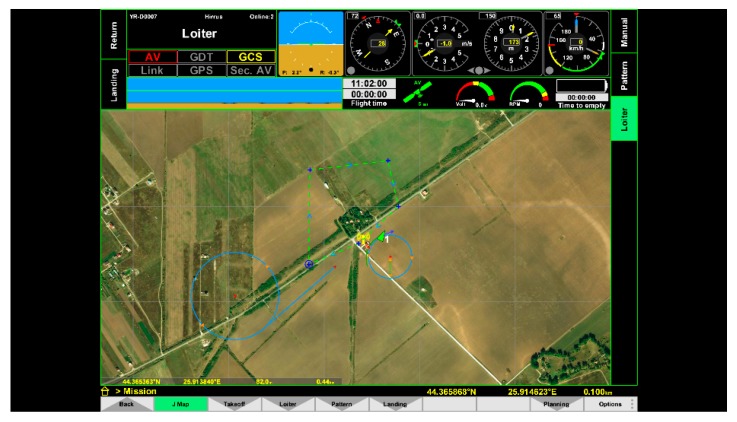
Loiter mode (tracking circles = blue). Green arrow = UAV.

To simulate path tracking, the NMEA (National Marine Electronics Association) Generator was used [46] (Figure 11). The path tracking, both in pattern mode (piecewise linear trajectory) and in loiter mode (circles around base stations), was simulated (Figure 12 and Figure 13).

### 3.2. Sensor Placement and Parameter Maps

UAV path planning revolves around optimizing the data collection from the cluster head with the constraint of limited mobility and hovering ability of fixed-wing type airborne platforms. To this extent, before the UAV is scheduled to visit the area, all local measurement have to be collected from the WSN at the cluster head, filtered, and aggregated, while only uploading, for example, the consensus values, confidence intervals, and outcomes of event detection and embedded alerting mechanisms.

**Figure 14:**
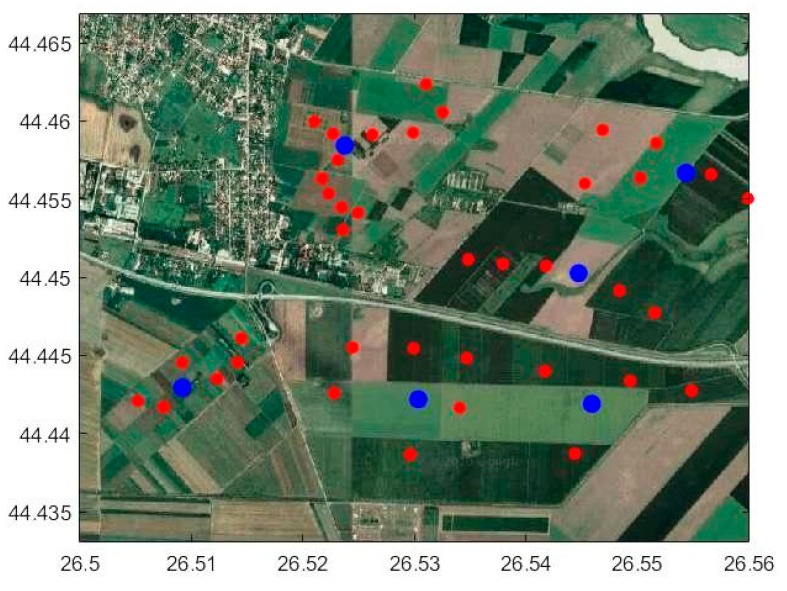
Study area with the corresponding sensor nodes (red disks) and cluster heads (blue disks).

The practical experiments at the ground sensor network level have used a sensor node deployment similar to the layout in Figure 14. In total, there are 45 nodes deployed in the field on various experimental parcels from our agronomical research institute partner. Among these nodes, six of them have the cluster head role for local collection of the sensor measurement from the neighboring nodes, as well as increased capabilities in terms of data processing, storage, and energy resources, e.g., solar panel, larger batteries, and high gain antennas for more robust operation. These are listed as blue disks in the figure, and their selection is based on the geographical coverage conditions and installation constraints.

**Figure 15:**
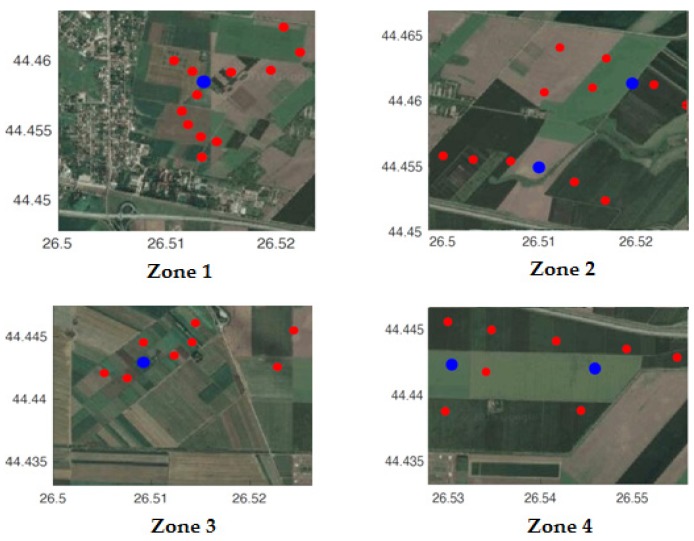
Location of nodes in four zones.

In Figure 15, a further split of the wireless sensor network is performed according to four interest zones (Zone 1–Zone 4) in the agricultural experimental area. Zone 1 contains one cluster head and 12 sensor nodes. Zone 4 contains one cluster head and six sensor nodes. For increased reliability of the data collection, in Zone 2 and Zone 3, two cluster heads are installed, with two patches of six and five sensor nodes, respectively, in the first case and two patches of six and four sensor nodes in the latter.

**Figure 16:**
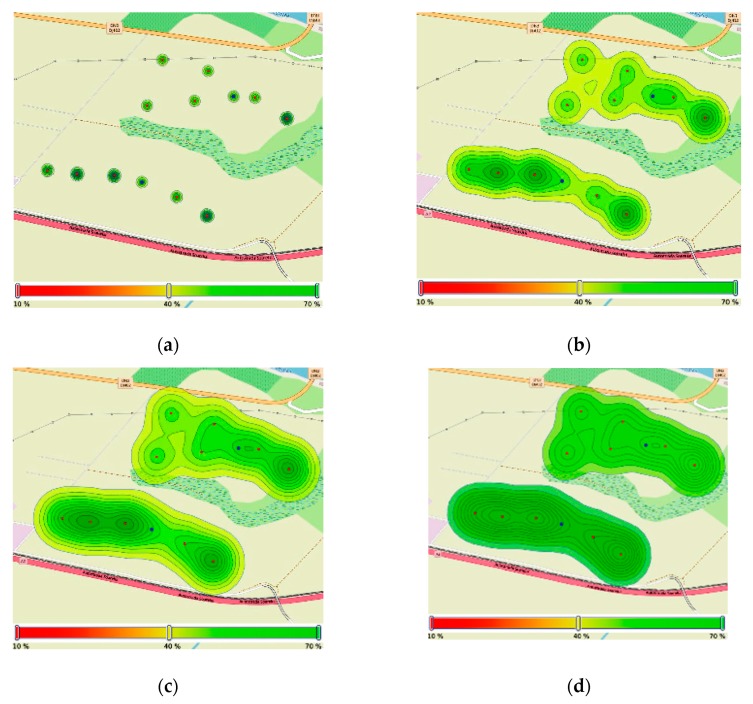
Soil moisture map in Zone 2, before and after consensus: (**a**) location of soil moisture sensors; (**b**) soil moisture map after 10 iterations; (**c**) soil moisture map after 20 iterations; and (**d**) soil moisture map after 30 iterations.

Based on the discussed deployment layout in the field, we present the coverage maps from the initial values for two parameters and their progression based on the implementation of the distributed agreement algorithm. In Figure 16a, the initial soil moisture values are presented. As the consensus algorithm advances in 10, 20, and 30 iterations, the coverage map is formed with increasing confidence on the joint agreement value after subsequent message exchanges. The final agreement value is stored at the cluster head to ultimately inform the decision process of the local conditions for irrigation actuation—the sensing density, in our case, is larger than the granularity of the irrigation system, which requires an average model based on the local geographical conditions.

**Figure 17:**
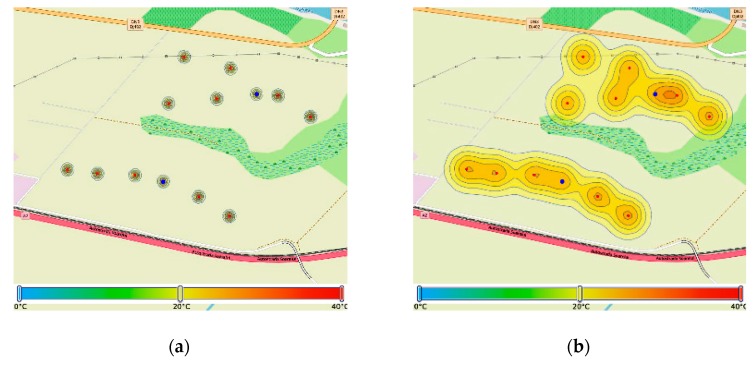

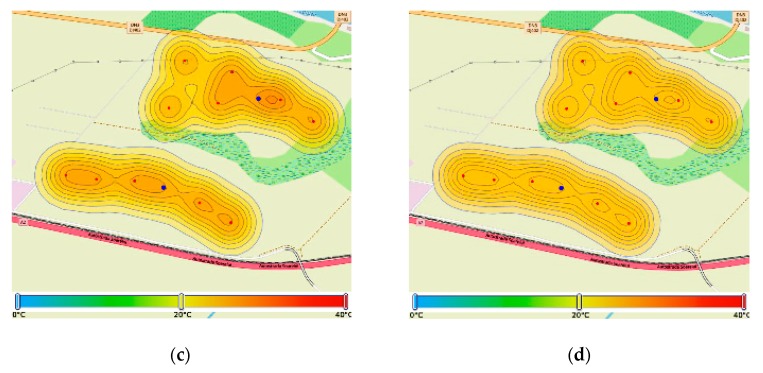
Temperature map in Zone 2, before and after consensus: (**a**) location of temperature sensors; (**b**) temperature map after 10 iterations; (**c**) temperature map after 20 iterations; and (**d**) temperature map after 30 iterations.

In a similar manner as for the soil moisture parameter, Figure 17 reports the initial values and the consensus progression for the air temperature parameter for Zone 2. The approach is repeated for all the parameters that can be sensed in the field. The sampling time is adapted to the process dynamics, as well as to previously reported events or external influences, e.g., weather changes, season, and expert input regarding field conditions.

### 3.3. Data Processing Results

As previously discussed, the primary local distributed agreement is based on consensus among the clustered sensing nodes. This allows the nodes to have a unitary representation of the measurements, under the assumption of limited variance in the geographical sensing area for one cluster. The parameters that are sampled by the nodes include: air temperature, relative humidity, soil temperature, soil moisture, and solar radiation.

**Figure 18:**
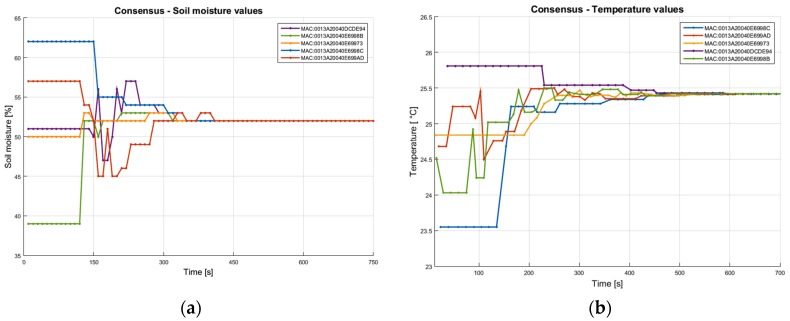
Consensus results for: (**a**) soil moisture and (**b**) air temperature.

Figure 18 illustrates the consensus results for two parameters: soil moisture and air temperature in a cluster of five TelosB sensor nodes. These are obtained through simulation in a Contiki/COOJA network environment starting from ground-collected values. The main insight provided by this result is in the analysis of the convergence time and convergence values in conjunction with fixed or dynamic tuning parameters. More specifically, by adjusting the communication frequency and weighting the consensus algorithm based on the sensor location and confidence levels, we can guide the algorithm with expert knowledge. This can result in acceleration of the process or in more reliable consensus values.

Once local agreement has been established, relevant data extraction is performed at the cluster head by means of the SAX method. In this case, we present the outcome for running the algorithm on a data sample of around 10 days, with the consensus values stored at 30-min intervals at one cluster head (Table 2). The variations in the SAX string length correspond to the parameterization of the method in terms of the number of segments to divide the input time series into (nseg) and the alphabet size, i.e., the discrete threshold levels numbers for classifying the processed values (alphabet_size). The number of samples of the input data is 490, for nseg = 20, corresponding to half daily patterns this is truncated to 480 as the total length of the time series must be divisible with the number of segments. Inputs are z-normalized for the computation of the assigned label. Data were collected in mid-July 2018.

The proposed relevant data extraction methods were evaluated from a comparative standpoint regarding the ratio between the volume of data and the data relevance. For a set of measurements, for air temperature monitoring, acquired for 10 days, 502 data points were validated and stored, totaling 2.008 kBytes. This raw data set was used for three relevant data extraction methods; the results are presented below.

**Figure 19:**
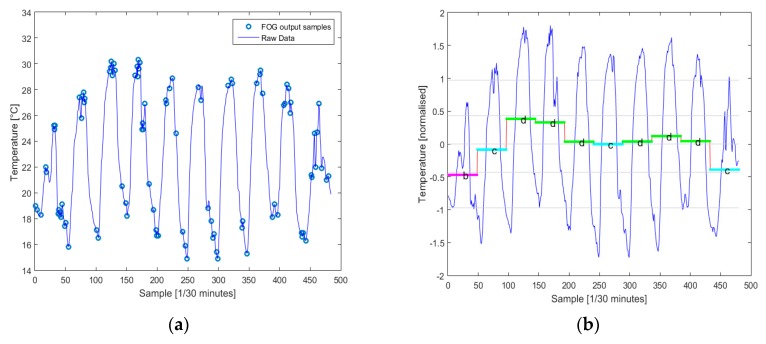

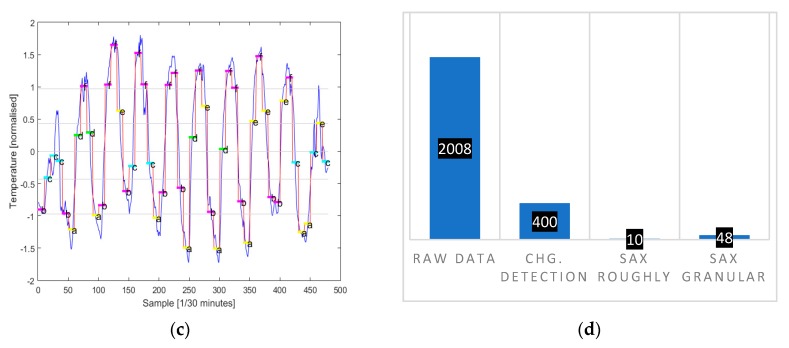
Relevant data extraction: (**a**) change detection method; (**b**) SAX algorithm–Roughly; (**c**) SAX algorithm–Granular; and (**d**) comparative representation of data sizes achieved using the proposed relevant data extraction methods.

Figure 19 illustrates a total of 98 relevant points extracted through the Fog computing algorithm based on change detection approach. Considering the common size of 4 bytes for floating point values, a total of approximately 400 bytes needs to be uploaded (excluding the proposed protocol frame).

For the symbolic aggregation method, two tests were performed, for two parameterizations of the SAX algorithm at opposite poles. First, Figure 19 illustrates the results for SAX algorithm adjusted for a rough representation of the time series; thus, a number of 10 characters is extracted. Considering the common size of one byte for ASCII character representation, a total of 40 bytes needs to be uploaded. Secondly, for granular SAX, Figure 19 illustrates a total of 48 points, thus totaling of 48 bytes.

## 4. Discussion

The paper represents a significant extension of Reference [47] with further details regarding the UAV trajectory tracking and implementation of the support path planning software interfaces and illustrative path planning examples. On the data processing and deployment of the ground sensor network, the results are further elaborated upon with coverage maps, improved consensus, and relevant data extraction results. The two-stage data processing methodology presented in this paper includes a consensus algorithm for distributed agreement for sensor node patches deployed in the field alongside a relevant data extraction step based on the consensus results. The first stage is intended to ensure agreement of all the data collection entities upon the measured parameters, as well as to increase data quality by limiting the effect of sending upstream erroneous sensor readings. The second stage aims to optimize the data collection time at the interface between the cluster head and the UAV acting as a data mule. Based on the compressed representation of SAX segments, the results can be expanded and further processed at the decision level, in the cloud. At the higher abstract layer in the cloud, the results presented in Table 2 can be interpreted using state-of-the-art text analytics tools. This is useful for quantitative assessment of univariate sequences, as well as correlations between multivariate string series. The character frequencies and recurring subsequences for certain parameters might be indicators for evolving phenomena at the ground level.

Potential drawbacks of the integrated system are related to the increased complexity for multi-level data processing, communication, and interoperability constraints between the aerial platform and the ground sensors. Increased administrative requirements have to be complied with, e.g., approving flight plans for each UAV mission, along with maintenance requirements that can stem from outdoor deployment of the nodes. We consider, however, that the benefits outweigh the discussed drawbacks of such a system.

## 5. Conclusions

The paper illustrated a case study for collaborative UAV–WSN operation in large scale monitoring for precision agriculture. The algorithms, techniques, and tools to enable seamless interoperability between the two domains are illustrated. Key contributions are argued in the design of optimized trajectories for UAV-enabled field data collection and for in-network data processing that allows efficient use of limited ground sensor network resources. Particularly, we propose combined segment and loiter tracking modes which balance between path length and time spent in the neighborhood of a cluster head. By passing the raw sensor readings through multiple hierarchical data processing steps, the quality of the extracted information is increased, as well as its timeliness, given the fact that reduced communication burden allows lower network-wide latency for decision-making. The role of the UAV platform is critical to support large scale monitoring and data collection applications in precision agriculture as it reduces the reliance of third-party communication and computing infrastructure that might not be readily available in the field or pose increased costs.

Extensive field evaluation is planned for validation of the impact of such a system for crop management. The main challenges for such a collaborative system are the following: sensing covering, communication covering by the hybrid UAV–ground WSN system, energy efficiency, and computing efficiency.

## Figures and Tables

**Table 1:** Processing levels.

Level	Content
Field	Sensors (SNs)
Edge computing	Cluster heads (CHs), UAV
Cloud computing	Cloud
Data interpretation	User server

**Table 2:** Resulting Symbolic Aggregate Approximation (SAX) strings on consensus data.

SAX Parameters	Solar Radiation	Air Temperature	Soil Temperature	Relative Humidity
nseg = 10 alphabet size = 4	bcccbccccb	bbcccbcccb	aabdccccdc	cccbbbbbbc
nseg = 10 alphabet size = 6	cdddcddddc	bcdddcdddc	aaceeddded	eddcccccce
nseg = 20 alphabet size = 4	bbbcbcbcbcbcbdbdbdab	abacbdbdadadadadbdac	aaaaaccdcccccccccdcb	dcdbdacadacadadacadc
nseg = 20 alphabet size = 6	bccdcecdbecebebebebc	bcbebfcfbebeafbfbead	aaabbdeeeededdddeeec	edebebebeaeaeaeaebed
